# Cytoplasmic Expression of p16 Is Associated with Carcinoma Breast: It Is Not an Artifact

**DOI:** 10.30699/IJP.2024.2006691.3186

**Published:** 2024-02-15

**Authors:** Sudipta Naskar, Nadeem Tanveer, Sonal Sharma, Navneet Kaur

**Affiliations:** 1 *Department of Pathology, University College of Medical Sciences & GTB Hospital, Delhi, India*; 2 *Department of Surgery, University College of Medical Sciences, Dilshad Garden, Delhi, India*

**Keywords:** Breast carcinoma, Comparison, Cytoplasmic p16, Fibroadenoma, p16 immunohistochemistry

## Abstract

**Background & Objective:**

p16 has different roles in the nuclear and cytoplasmic locations. The nuclear localization of the p16 protein explains its role in cell cycle regulation. Cytoplasmic expression was considered an artifact in the initial years, but there is evidence to prove that cytoplasmic localization is real and that p16 has different roles in the nuclear and cytoplasmic locations. We aimed to study the immunoexpression of p16 protein in the nuclear and cytoplasmic locations of the epithelial and stromal compartments of fibroadenoma, invasive breast carcinoma, and a select number of phyllodes tumors.

**Methods:**

The study included a total of 107 patients, comprising 51 cases of invasive breast carcinoma, 51 cases of fibroadenoma, 4 cases of benign phyllodes tumors, and 1 case of lobular carcinoma in situ (LCIS). The p16 immunohistochemistry was evaluated for nuclear and cytoplasmic localization in the epithelial and stromal compartments of the tumors.

**Results:**

Of the 51 fibroadenoma cases, 23 showed strong nuclear p16 epithelial expression, but no case showed cytoplasmic expression. In 19/51 cases, stromal cells also showed strong p16 nuclear expression. Moderate stromal p16 expression was observed in 3 out of 4 cases of benign phyllodes. Out of the 51 cases of invasive carcinoma, 31 showed moderate to strong nuclear p16 immunopositivity, while 27 cases exhibited cytoplasmic p16 expression. We found a statistically significant correlation between moderate to strong nuclear p16 immunoexpression and the molecular subtype of breast carcinoma.

**Conclusion:**

The cytoplasmic localization of p16 immunohistochemistry is not seen in epithelial components of fibroadenoma, while it is seen frequently in breast carcinoma. Nuclear p16 expression has a statistically significant correlation with molecular subtypes of breast carcinoma.

## Introduction

p16 protein is a cyclin-dependent kinase (CDK) inhibitor that is encoded by the tumor suppressor gene p16INK4A. This gene is located on chromosome 9p21.3. p16 inhibits phosphorylation of the retinoblastoma (RB) protein by inhibiting CDK 4/6, putting a brake on cell cycle progression.

It is a versatile protein that plays a key role in maintaining a balance between aging, tumor suppression, and cell cycle arrest (1). Cellular proliferation is essential for tissue homeostasis, and its deregulation leads to aging and promotes tumorigenesis. p16 protein blocks cellular proliferation and its prolonged activation causes cellular senescence. 

Aberrations in the RB pathway contribute to the elevated p16 immunoexpression in tumors. However, the RB pathway can be deregulated by a variety of mechanisms like viral oncogene expression, RB1 mutations, and cyclin gene amplification (1). p16 immunoexpression in oropharyngeal squamous cell carcinoma is a marker for human papillomavirus (HPV) infection and a predictor of better therapeutic response and survival (2).

Since the p16 protein plays an important role in aging, apoptosis, and carcinogenesis, its pattern of positivity varies with the parent organ and the tumor under study. Immunohistochemical expression of the p16 protein has been documented in the endometrium, ductal epithelium of breast, epidermis, salivary gland tissue, metaplastic endocervical cells, and Sertoli and Leydig cells. In infants, the expression is limited to the thymus, which is the only organ destined to undergo early senescence (3).

Immunoexpression of p16 protein has been reported in a significant proportion of triple-negative breast cancer (TNBC) and portends a poor prognosis (4). Recent studies have provided insights into the possible role of stromal p16 expression in tumorigenesis. The non-proliferative stroma in cases of ductal carcinoma in situ (DCIS) shows an increased p16 expression in a significant proportion of cases, and this is strongly correlated with disease recurrence (5). The peritumoral stromal expression of p16 has been reported to be significantly increased in endometrial adenocarcinoma and may have a role in tumorigenesis (6).

The stromal expression of p16 in phyllodes tumors has been correlated with increasing grade (7).

The nuclear localization of the p16 protein explains its role in cell cycle regulation. However, while evaluating p16 immunohistochemistry in tumors, pathologists look at nuclear and cytoplasmic expression. The cytoplasmic expression was considered an artifact in the initial years, but now we have enough evidence to prove that cytoplasmic localization is real and that p16 has different roles in nuclear and cytoplasmic locations (8).

This study aimed to a comprehensive evaluation of the immunoexpression of p16 protein in the nuclear and cytoplasmic locations of the epithelial and stromal compartments of fibroadenoma, invasive breast carcinoma, and a select number of phyllodes tumors.

## Material and Methods

This prospective study was conducted over 2 years (November 2018 to October 2020) at a tertiary care hospital in north India. To estimate the difference in the proportion of p16 expression of moderate to strong in about 40% of carcinoma breast cases and 6% of fibroadenoma cases, with an alpha level of 5 (0.05) and a power of 80%, a minimum sample size of 31 cases in each group was necessary to achieve statistical significance. A total of 107 patients who underwent a lumpectomy, modified radical mastectomy, or simple mastectomy for breast tumors were included in this study as follows:

1. Fifty-one cases of invasive breast carcinoma

- Age <35 years (17 cases)
- Age 35-50 years (17 cases)
- Age >50 years (17 cases)

1. Fifty-one cases of fibroadenoma

- Age <25 years (17 cases)
- Age 25-30 years (17 cases)
- Age >30 years (17 cases)

1. Four cases of benign phyllodes tumor
2. One case of LCIS

To remove the age-related bias in carcinoma and fibroadenoma cases, patients were divided into 3 age groups as mentioned above, and an equal number of cases were taken in each group randomly. 

The clinical details of the patients were taken from the histopathology requisition forms and patient records. The patients who had received neoadjuvant chemotherapy were excluded from the study. Cases with equivocal Her2/neu immunostaining were excluded from the study due to a lack of the in-house fluorescence in situ hybridization test for Her2.

Tissue samples were fixed in 10% buffered formalin for 24 hr before processing and embedding. The hematoxylin and eosin-stained sections of invasive carcinoma cases were reviewed for histological type, grade (Nottingham modification of Bloom Richardson Grading system), presence or absence of in situ component, and lymph node metastasis.

One representative block of tumor tissue from the advancing front of the viable tumor was chosen and routinely processed for immunohistochemistry. The different antibodies and their dilutions used are summarized in Table 1. The positive control used for p16 was a known p16 immunopositive case of high-grade squamous intraepithelial lesion of the cervix.

Estrogen receptor (ER), Progesterone receptor (PR), and HER2/neu immunostaining were done for all the carcinoma cases (Table 1). Known ER, PR, and Her2/neu positive carcinoma breast cases were taken as positive controls.

ER/PR positivity was defined according to the ASCO/CAP guidelines (>1% tumor cells positive), and immunoexpression for HER2 was assessed according to recent US Food and Drug Administration (FDA)-approved guidelines (9).

Expression of p16 was assessed at the invasive front of the tumor. Scoring of p16 was done using a semi-quantitative scoring system (10). Intensity (I) was given a score of 0 (negative), 1 (weak), 2 (moderate), and 3 (strong) and was multiplied by the percentage (P) of positive cells given a score of 0 (<1%), 1 (1% to 10%), 2 (11% to 50%), 3 (51% to 90%), and 4 (>90%). 

The combined score (I x P) was graded as weak (1), moderate (2 to 4), and strong (6 or more).

For fibroadenoma and phyllodes tumor cases, only p16 immunostaining was performed, and p16 score was assessed.

The immunostaining was scored separately for nuclear, cytoplasmic, epithelial, and stromal compartments.

For statistical analysis, data were imported and merged in SPSS version 22 (SPSS Inc., Chicago, IL., USA). Spearman correlation was used to see any significant correlation between 2 independent variables for the carcinoma and fibroadenoma cases. Fisher's exact test was used to compare p16 expression. A P-value of ≤ 0.05 was considered statistically significant.

The study was conducted on the archival blocks in accordance with the principles of the Declaration of Helsinki after obtaining approval from the Institute Ethics Committee.

**Table 1:** Protocol for the antibodies used for immunohistochemistry.

Antibody	Company	Clone	Source	Dilution	Antigen retrieval Buffer	Antigen retrieval (PC)	Incubation	Detection
ER	Thermo-Fisher	SP-1	Rmab	PD	Tris-EDTA	95˚C pH9.0 x 300	4˚Cx18 Hours	PolyDetector DAB HRP Brown (Bio SB) Substrate - Chromogen
PR	Thermo-Fisher	SP-2	Rmab	PD	Tris-EDTA	95˚C pH9.0 x 300	4˚Cx18 Hours	PolyDetector DAB HRP Brown (Bio SB)
HER-2/*neu*	Thermo-Fisher	SP-3	Rmab	PD	Tris-EDTA	95˚C pH9.0x300	4˚Cx18 Hours	PolyDetector DAB HRP Brown (Bio SB)
p16	Invitrogen (Thermo-Fisher)	5A8A4	Mmab	1:200	Citrate	95˚C pH6.0 x300	4˚Cx18 Hours	PolyDetector DAB HRP Brown (Bio SB)

## Results

Of the 51 invasive carcinomas, 49 were IDC-NST type, 1 was IDC with medullary-like features, and 1 was tubulolobular carcinoma. Fifteen cases (29.4%) were triple-negative, 19 cases (37.3%) were Grade 3, and 23 cases (45.1%) were Grade 2. We subdivided the carcinoma case into 3 molecular subtypes: luminal (ER and/or PR positive, HER2 ±), HER2 enriched (ER and PR negative, HER2 positive), and triple-negative (ER, PR, and HER2 all negative). However, the Her2 equivocal cases on immunohistochemistry were excluded due to a lack of fluorescence in situ hybridization (FISH) analysis.

**Fig. 1:**
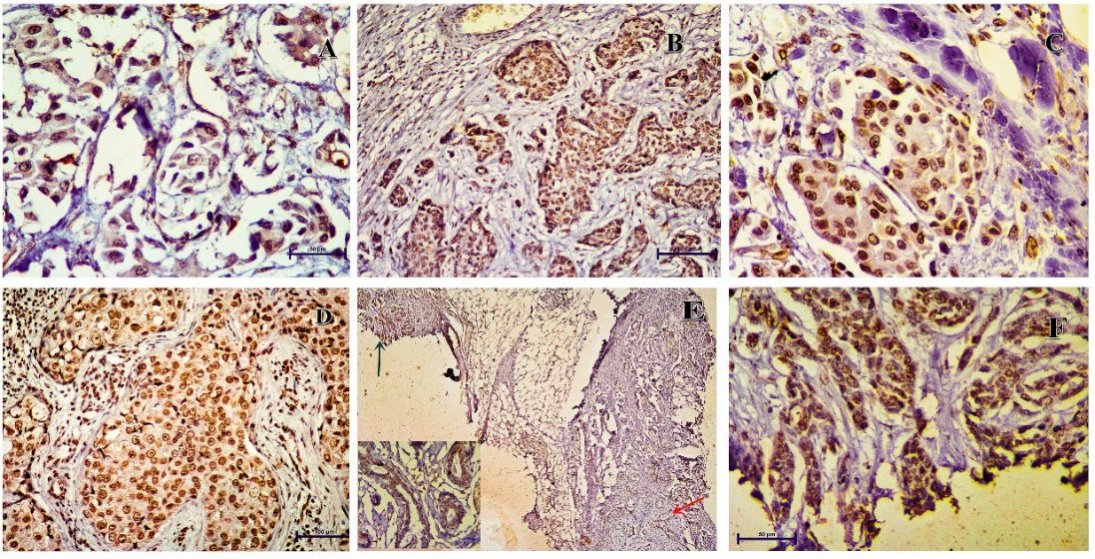
p16 expression in the carcinoma cases. A and C (400x) - Invasive ductal carcinoma (IDC) grade 2 with strong nuclear and moderate cytoplasmic immunopositivity. B (200x)-IDC grade 3 showing moderate nuclear and cytoplasmic positivity. D (400x) A case of IDC with medullary features showing moderate nuclear and cytoplasmic positivity. Few lymphocytes are also showing p16 nuclear immunopositivity. E (100x) IDC grade 2 and adjacent ductal carcinoma in situ (Green arrow) both show moderate nuclear and cytoplasmic immunopositivity. F (400x) Triple-Negative Breast Carcinoma showing strong nuclear and moderate cytoplasmic immunopositivity.

**p16 Expression in Carcinoma Cases (** Table 2 **and** Figure 1 **)**

**Fig. 2:**
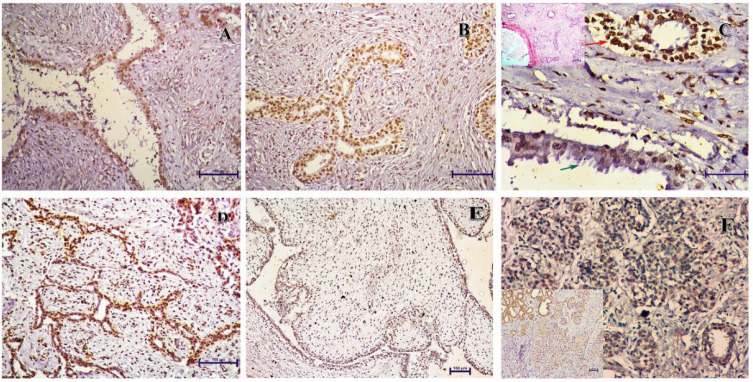
Immunohistochemistry for p16 in the fibroadenoma cases: A (400x) Fibroadenoma with moderate nuclear and cytoplasmic immunopositivity. B (400x) Fibroadenoma with strong nuclear and moderate cytoplasmic expression. C (400x) Weak expression in apocrine metaplasia (green arrow) and strong expression in other areas (red arrow) of fibroadenoma. D (400x) Moderate stromal immunopositivity in fibroadenoma. E (100x) Benign Phyllodes tumor with strong nuclear and moderate stromal immunopositivity. F (400x) Lobular carcinoma in situ case showing weak nuclear and cytoplasmic immunopositivity.

A total of 43/51 cases showed nuclear p16 immunopositivity and 27/51 cases showed cytoplasmic p16 expression. Fifteen cases showed moderate to strong cytoplasmic immunopositivity, and 22 cases showed strong nuclear p16 expression. Among TNBCs, 11/15 cases showed strong nuclear expression (*P*=0.020), and 10 of them also had cytoplasmic p16 expression (Table 3). Nine out of the 19 cases of Grade 3 carcinomas and 11 out of the 23 cases of Grade 2 carcinomas showed strong nuclear p16 expression. In the LCIS case, p16 expression was weak in the tumor and the surrounding normal breast (Figure 2F). In most of the cases, the stroma was weakly positive or negative. However, we did not find any cases with diffuse block-type positivity of p16. 

There was a strong correlation between the molecular subtype of the tumor and p16 nuclear expression (*P*=0.020). The correlation with other parameters like age, grade, nodal status, and tumor stage did not reach statistical significance. Among the 24 cases that did not show any cytoplasmic expression, 13 were of the luminal subtype; however, the correlation between cytoplasmic expression of p16 and molecular subtype was not statistically significant (*P*=0.114).

**p16 Expression in Fibroadenoma Cases (** Table 3 **and** Figure 2 **)**

Of the 51 cases, 23 showed strong nuclear p16 expression, but no case of fibroadenoma showed cytoplasmic p16 expression. In 19 cases, stromal cells also showed strong p16 expression (nuclear only). In tumors of less than 30 mm, 16/29 cases showed strong p16 expression in the epithelial component, and 15/29 cases had strong stromal p16 expression; however, in tumors of >30 mm, 14/22 cases had either weak or negative p16 stromal expression (*P*=0.005). In >30 years, 11/17 patients showed strong epithelial p16 expression (Table 3). One fibroadenoma had apocrine changes, which had weak p16 expression in comparison to strong p16 expression in the rest of the tumor (Figure 2C). There was a statistically significant correlation between stromal nuclear positivity and the size (less than 3 cm) of the tumor.

**Table 2:** p16 expression in breast carcinoma cases and its correlation with the molecular subtype, age, histological grade, nodal status, and tumor size.

		Nuclear p16 Expression in Carcinoma				*P*	Cytoplasmic p16 expression in carcinoma				*P*	Stromal p16 expression in carcinoma				*P*
**Negative**	**Weak**	**Moderate**	**Strong**	**Negative**	**Weak**	**Moderate**	**Strong**	**Negative**	**Weak**	**Moderate**	**Strong**
Molecular Subtyping	Luminal	6	4	7	6	0.020	13	6	2	2	0.114	11	8	2	2	0.696
	Her2	0	7	1	5		6	3	2	2		2	10	1	0	
	TN	2	1	1	11		5	3	6	1		7	4	2	2	
Patient's Age	≤35 Years	2	1	4	10	0.166	5	6	4	2	0.428	8	6	3	0	0.084
	35 to 50	4	5	2	6		11	2	3	1		9	6	1	1	
	> 50 Years	2	6	3	6		8	4	3	2		3	10	1	3	
Histological Grade	One	3	2	2	2	0.215	5	2	2	0	0.825	3	5	1	0	0.546
	Two	3	5	4	11		10	5	5	3		9	8	3	3	
	Three	2	5	3	9		9	5	3	2		8	9	1	1	
Nodal Status	Stage N0	2	2	4	8	0.515	7	4	3	2	0.599	11	4	1	0	0.015
	Stage N1	1	5	3	4		7	3	3	0		2	9	1	1	
	Stage N2	4	2	2	6		7	2	3	2		6	3	3	2	
	Stage N3	1	3	0	4		3	3	1	1		1	6	0	1	
Pathological Stage	pT1	2	1	2	3	0.138	4	1	1	2	0.599	5	1	1	1	0.128
	pT2	4	8	4	9		13	6	6	0		10	12	3	0	
	pT3	2	3	2	5		5	3	1	3		4	6	1	1	
	pT4	0	0	1	5		2	2	2	0		1	3	0	2	

**Table 3:** p16 expression in the fibroadenoma cases.

		Nuclear p16 Expression in epithelial component			*P*	Stromal p16 Expression (nuclear)				*P*
		Weak	Moderate	Strong		Negative	Weak	Moderate	Strong	
Age Group	< 25 Years	10	4	3	0.007	7	5	2	3	0.038
	25 to 30 Years	5	3	9		5	1	4	7	
	>30 Years	4	2	11		3	4	1	9	
Tumor size	< 30 mm	9	4	16	0.139	4	7	3	15	0.005
	> 30 mm	10	5	7		11	3	4	4	

**p16 Expression in the Phyllodes Tumor Cases (** Table 4 **)**

All samples were histologically benign phyllodes tumors. Three of these showed strong nuclear p16 expression in the epithelial component, and 1 showed weak cytoplasmic expression. Three showed moderate stromal p16 expression, and 1 showed weak p16 expression.

**Coexisting Fibroadenoma and Carcinoma**

Two triple-negative IDC-NST had coexisting fibroadenoma at the advancing front of carcinoma. Both fibroadenomas were <30 mm. In both carcinoma components, there was moderate cytoplasmic p16 expression, but the fibroadenoma part did not show any cytoplasmic expression; however, nuclear p16 expression was strong in both carcinoma and fibroadenoma parts.

**Table 4:** p16 expression in the benign phyllodes cases

Serial	Case no	Age	Epithelial p16_Expression	Stromal p16_Expression	Cytoplasmic p16 Expression (epithelial)
1	3517	45	Strong	Moderate	Negative
2	3723	42	Strong	Moderate	Weak
3	14456	22	Strong	Moderate	Negative
4	2479	40	Weak	Weak	Negative

## Discussion

As a cell cycle regulatory protein, the main site of action of p16 is inside the nucleus, where it binds with CDK 4/6 and stops cells from progressing from G1 to the S phase. After translation and post-translational modifications, the p16 protein is transported inside the nucleus by the nuclear transport system. However, this translocation can be impaired if p16 binds to another protein, resulting in the formation of a macromolecule and subsequent intracytoplasmic accumulation of p16 (8). Expression of p16 has been associated with an aggressive phenotype of breast carcinoma (11). In benign neoplasms (like melanocytic nevi and benign nerve sheath tumors), tumor cells are arrested in the G_0_ phase and undergo senescence due to overexpression of the p16 protein. Overexpression of p16 helps in putting a brake on cell proliferation. However, malignant counterparts of both tumors, i.e., melanoma and malignant peripheral nerve sheath tumor, are immunohistochemically negative for p16 (12, 13).

We did not find any carcinoma cases with p16 immunopositivity restricted to the cytoplasm. All the cases with moderate to strong cytoplasmic expression of p16 also had a strong or moderate nuclear p16 immunopositivity (Figure 1). For fibroadenoma cases, the immunopositivity was restricted to the nucleus of both the epithelial and stromal components. None of the cases showed any cytoplasmic immunopositivity. Similar findings have been reported by Feriancová *et al.* (14).

Due to its role in senescence and aging, it is anticipated that the immunoexpression of p16 would increase with age. p16 is being evaluated as a possible biomarker for the aging effect of various chemotherapy regimens (15). However, its expression in fibroadenoma and carcinoma did not correlate with the age of the patient. This may be because neoplastic processes tend to override the senescence-induced p16 expression. Since p16 is linked to senescence, we designed the study in such a way so that tumor specimens from different age groups are included, and there is no age bias in patient selection.

The expression of p16 and other cell cycle regulators has been reported to be higher at the invasive front of carcinoma (16). Hence, to ensure uniformity in the p16 evaluation, the blocks representing the invasive tumor front of breast carcinoma were chosen for p16 immunohistochemistry. This is an improvement over previous studies of p16 in breast carcinoma, where the criterion for choosing the block has not been specified (10, 11).

Our study found a statistically significant correlation between the molecular subtype of breast carcinoma and nuclear p16 expression. However, no statistically significant correlation was found between cytoplasmic intensity taken separately and the molecular subtype. These findings are similar to previous studies; however, the nuclear and cytoplasmic expressions were not evaluated separately in these studies (17). One limitation of our study was the non-availability of FISH for Her2/neu. The cases equivocal for Her2/neu staining on immunohistochemistry were excluded from the analysis.

The stromal immunopositivity for p16 in combination with pRB has been suggested to be a possible marker for differentiating benign phyllodes from fibroadenoma (18). However, we found moderate to strong p16 stromal immunoexpression in 26/51 fibroadenomas and 3/4 benign phyllodes. Hence, there is considerable overlap between these 2 entities for p16 stromal expression (Figures 2D and 2E). Interestingly, 3 out of 4 phyllodes tumors also showed strong epithelial nuclear immunopositivity, and only 1 case showed weak cytoplasmic immunopositivity. Cytoplasmic immunopositivity was absent in all 51 fibroadenomas (Figures 2A, 2B, and 2C).

Our findings are consistent with those of Di Vinci *et al.*, reporting that the pattern of positivity in fibroadenoma is almost always nuclear, while cytoplasmic immunopositivity is frequently seen in breast carcinoma (19). They reported that the positivity could be rarely restricted to the cytoplasm only in carcinoma cases; however, we did not find any such case. In their study, the only fibroadenoma case having weak cytoplasmic immunopositivity had features reminiscent of benign phyllodes. We did not find any cytoplasmic immunopositivity in fibroadenoma; however, 1 case of benign phyllodes showed weak cytoplasmic immunopositivity.

The stromal immunopositivity in fibroadenoma was more in the smaller (less than 3 cm) tumors, suggesting a role of stromal p16 in arresting the growth of fibroadenoma.

Moderate to strong stromal immunopositivity was observed in 9/51 cases of breast carcinoma and was not correlated with molecular subtype, age, grade, lymph node metastasis, or T stage of the tumor. Thus, p16 stromal immunopositivity is an adverse prognostic indicator for DCIS but not for invasive carcinoma (5).

An interesting observation was the nuclear immunopositivity of a few tumor-infiltrating lymphocytes in the breast carcinoma cases for p16 (Figure 1D). This may be related to lymphocyte senescence or the role of p16 in T-cell apoptosis (20). Another finding was the weaker immunoexpression of p16 in apocrine metaplastic cells in fibroadenoma (Figure 2C). Previous studies have also reported weak to absent p16 expression in non-papillary apocrine metaplasia of the breast (21). This supports the hypothesis that there is lesser cellular unrest in these areas, making them less susceptible to malignancy. The pattern and intensity of p16 immunopositivity remained the same in the DCIS and invasive components of carcinoma cases (Figure 1E).

A few studies in the literature have evaluated the difference in nuclear and cytoplasmic expression of p16 in tumors (19, 22). As we gather more data on this theme for different tumors, our understanding of the myriad roles that this protein plays in different organs of the body will improve. There is a considerable difference of opinion as to whether nuclear/cytoplasmic or only nuclear localization of p16 should be considered positive in tumors. The justification for favoring one over the other is missing. The only way to solve this problem is to study benign and malignant tumors of each site and compare their expression. A recent study has described 14 interactors of cytoplasmic p16, facilitating nuclear shuttling in cervical squamous cell carcinoma (8). Based on our findings, p16 acts as a tumor suppressor gene in benign tumors like fibroadenoma. Its nuclear expression in fibroadenoma suggests that it prevents malignancy in the setting of the proliferation of epithelial and stromal components. However, in carcinoma cases, its cytoplasmic interactors prevent its translocation to the nucleus, and it is not able to suppress malignancy. However, these interactors are not very efficient in their function, and a considerable amount of p16 still escapes to the nucleus, resulting in combined nuclear and cytoplasmic immunoexpression. Of the 28 hormone receptor-negative cases, 17 expressed nuclear and cytoplasmic p16, suggesting that these may have some impact on cytoplasmic interactors and sequesters of p16.

Hu *et al.* demonstrated decreased nuclear p16 expression and increased cytoplasmic p16 expression in the malignant component of carcinoma ex pleomorphic adenoma of the salivary gland (23). The 2 fibroadenoma cases associated with invasive ductal carcinoma included in this study also mirrored these findings; cytoplasmic expression was noted only in the carcinoma and not in fibroadenoma. Hence, the p16 immunostaining pattern in fibroadenoma cannot be used to predict the chances of developing concurrent carcinoma.

Liu *et al.* showed that cytoplasmic expression of p16 in gastric carcinoma cases was associated with increased invasiveness, but interestingly, it was also associated with decreased metastatic potential (24). In another study by Arifin *et al.*, cytoplasmic expression of p16 was associated with unfavorable prognosis in high-grade astrocytoma patients (25).

Recently, a novel suicide gene therapy has been attempted for tumors exhibiting elevated p16 protein expression, and promising results have been observed in animal models (26). Targeted therapy for p16 is also on the anvil (27). According to a recent study, cytoplasmic expression of p16 can act as both a prognostic and predictive factor in head and neck squamous cell carcinoma (28). Similarly, few studies on breast cancers have shown that p16 can be a prognostic and predictive factor (29,30), and its cytoplasmic localization is an indicator of tumor progression (31). 

A few limitations of this study are noteworthy. The sample size is limited. Other immunomarkers related to the p16 pathway (like pRB and Ki67) were not evaluated. Follow-up data of the patients were not available. However, we hope that future studies on cytoplasmic interactors of p16 in breast neoplasms will provide new insights.

## Conclusion

None.
